# Monitoring of magmatic–hydrothermal system by noble gas and carbon isotopic compositions of fumarolic gases

**DOI:** 10.1038/s41598-022-22280-3

**Published:** 2022-11-21

**Authors:** Tomoya Obase, Hirochika Sumino, Kotaro Toyama, Kaori Kawana, Kohei Yamane, Muga Yaguchi, Akihiko Terada, Takeshi Ohba

**Affiliations:** 1 Department of General Systems Studies, Graduate School of Arts and Sciences, The University of Tokyo, 3-8-1 Komaba, Meguro, Tokyo, 153-0041 Japan; 2 Department of Earth and Planetary Sciences, Faculty of Science, Hokkaido University, Sapporo, Hokkaido 060-0810 Japan; 3 Research Center for Advanced Science and Technology, The University of Tokyo, 4-6-1 Komaba, Meguro, Tokyo, 153-0041 Japan; 4 Hot Springs Research Institute of Kanagawa Prefecture, Odawara, Kanagawa 250-0031 Japan; 5 Earth Surface System Research Center, Research Institute for Global Change, Japan Agency for Marine-Earth Science and Technology (JAMSTEC), Yokohama, Kanagawa 236-0001 Japan; 6 Meteorological Research Institute, Japan Meteorological Agency, 1-1 Nagamine, Tsukuba, Ibaraki 305-0052 Japan; 7 Volcanic Fluid Research Center, School of Science, Tokyo Institute of Technology, 2-12-1 Ookayama, Meguro, Tokyo, 152-8551 Japan; 8 Department of Chemistry, School of Science, Tokai University, 4-1-1 Kitakaname, Hiratsuka, Kanagawa 259-1291 Japan

**Keywords:** Natural hazards, Solid Earth sciences

## Abstract

We repeatedly measured isotopic compositions of noble gases and CO_2_ in volcanic gases sampled at six fumaroles around the Kusatsu-Shirane volcano (Japan) between 2014 and 2021 to detect variations reflecting recent volcanic activity. The synchronous increases in ^3^He/^4^He at some fumaroles suggest an increase in magmatic gas supply since 2018. The increase in magmatic gas supply is also supported by the temporal variations in ^3^He/CO_2_ ratios and carbon isotopic ratios of CO_2_. The ^3^He/^40^Ar* ratios (^40^Ar*: magmatic ^40^Ar) show significant increases in the period of high ^3^He/^4^He ratios. The temporal variation in ^3^He/^40^Ar* ratios may reflect changes in magma vesicularity. Therefore, the ^3^He/^40^Ar* ratio of fumarolic gases is a useful parameter to monitor the current state of degassing magma, which is essential for understanding the deep process of volcanic unrest and may contribute to identifying precursors of a future eruption. These results provide additional validation for the use of noble gas and carbon isotopic compositions of fumarolic gases for monitoring magmatic–hydrothermal systems.

## Introduction

The compositions of volcanic gases reflect deep to subsurface volcanic processes such as degassing from magma, incorporation of shallower components, and vapor–liquid separation^1^. Hence, volcanic gases provide direct information about a magmatic‒hydrothermal system beneath a volcano.

The ^3^He/^4^He ratios of volcanic gases (up to ~ 8 R_A_ in Japan^2^, where R_A_ denotes the atmospheric ^3^He/^4^He ratio of 1.4 × 10^−6^; ref.^3^) are sometimes lower than the ^3^He/^4^He ratio in the mantle (7–9 R_A_^4^) due to the incorporation of crustal He with a low ^3^He/^4^He ratio of ~ 0.01–0.02 R_A_^5^. The low crustal ^3^He/^4^He ratio is due to ^4^He production by the decay of U and Th. Since the proportion of magmatic and crustal He in volcanic gases may vary in response to magmatic gas flux, ^3^He/^4^He ratios might be useful for monitoring volcanic activity. For example, pre-eruptive ^3^He/^4^He ratio increases have been reported at some volcanoes, suggesting that an increase in magmatic He contribution could precede the eruptions^6–8^.

Some fractions of CO_2_ in fumarolic gases might be added by shallow processes after degassing from magma, such as decarbonation of crustal limestone^9,10^. The relative contributions of major CO_2_ sources in fumarolic gases can be estimated by combining the ^3^He/CO_2_ ratios and carbon isotopic ratios of CO_2_^11^. Although some physicochemical processes at shallow depths can modify both parameters, the spatial-temporal variations in ^3^He/CO_2_ ratios and carbon isotope ratios of CO_2_ have been used to monitor volcanic activities and understand the structures of magmatic‒hydrothermal systems^12–17^.

Some temporal variations in the relative abundances of volatile species in magmatic gases have been attributed to solubility-controlled fractionation that correlates with the vesicularity of degassing magma^13,15,16,18–20^. Since noble gases such as He and Ar are chemically inert, their abundance ratios are not affected by any chemical process. Thus, the temporal variations in magmatic ^3^He/^40^Ar* ratios (^40^Ar*: ^40^Ar corrected for the atmospheric contribution) in volcanic gases may reflect a change in the state of degassing magma.

**Figure 1:**
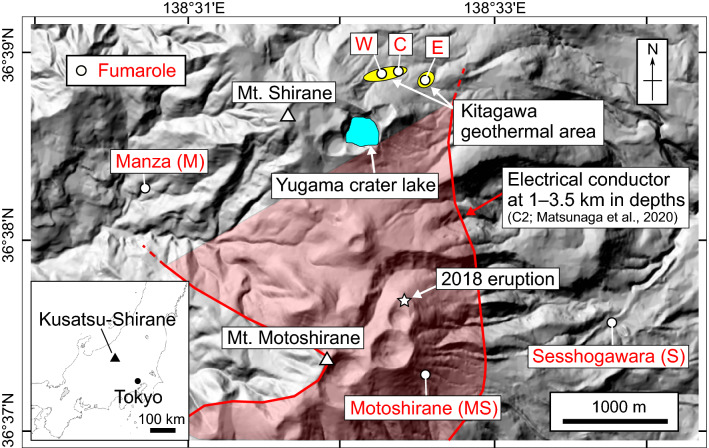
Map of the Kusatsu-Shirane volcano. White circles are the locations of fumaroles where samples were collected for this study. A white star indicates the location of the phreatic eruption on 23 January 2018. Yellow circles show the Kitagawa geothermal area located on the northern flank of Mt. Shirane. The red area indicates the horizontal extent of electrical conductor “C2”, which is probably a hydrothermal fluid reservoir beneath the Kusatsu-Shirane volcano^23^. The base map is from the website of the Geospatial Information Authority of Japan (https://maps.gsi.go.jp/).

To investigate the applicability of these isotopic parameters to monitoring the magmatic‒hydrothermal system of the Kusatsu-Shirane volcano, we have repeatedly measured the concentrations and isotopic compositions of noble gases and CO_2_ in fumarolic gases since 2014. Kusatsu-Shirane is an active stratovolcano that consists of three pyroclastic cones: Mt. Shirane, Mt. Ainomine, and Mt. Motoshirane, where phreatic eruptions frequently occur^21^. The presence of an electrically conductive structure at depths of 1–3.5 km broadly extending from beneath Mt. Shirane to Mt. Motoshirane has been detected by magnetotelluric surveys (Fig. 1), and this structure has been interpreted as a fluid reservoir supplying magmatic‒hydrothermal fluid to fumaroles and hot springs^22–24^. Because of its frequent eruptions and long-term multiparameter observations of volcanic activity^21,25–28^, Kusatsu-Shirane is one of the best case study fields for monitoring a magmatic‒hydrothermal system.

Fumarolic gases were repeatedly sampled at three fumaroles in the Kitagawa geothermal area (Kitagawa fumaroles W, C, and E), the Sesshogawara fumarole (S) on the eastern side of the volcano, the Manza fumarole (M) on the western side of the volcano, and the Motoshirane fumarole (MS) on the eastern side of Mt. Motoshirane (Fig. 1 and Supplementary Fig. S1). The sampling periods are between July 2015 and April 2021 for the W and E fumaroles, between October 2016 and April 2021 for the C fumarole, between October 2014 and April 2021 for the Sesshogawara fumarole, and between March 2018 and April 2021 for the Manza fumarole. The Motoshirane fumarolic gas was sampled on 11 August 2020.

In this paper, we discuss following topics based on the fumarolic gas compositions at the Kusatsu-Shirane volcano: (1) temporal variations in magmatic gas supply based on ^3^He/^4^He ratios; (2) CO_2_ sources feeding fumaroles based on spatial–temporal variations in ^3^He/CO_2_ ratios and carbon isotopic compositions; and (3) temporal variations in magma vesicularity based on ^3^He/^40^Ar* ratios.

## Results

### Temporal variations in ^3^He/^4^He ratios

The noble gas and CO_2_ concentrations, He, Ne, and Ar isotopic ratios, and ^13^C/^12^C ratios of CO_2_ in the fumarolic gas samples are listed in Supplementary Table S1. The ^13^C/^12^C ratios are shown in the delta (δ) notation as parts per thousand deviations (per mil, ‰) from the international Pee Dee Belemnite (PDB) standard. Five samples were collected as residual gases (R-gases) in Giggenbach-type bottles (see “Methods” section and Supplementary Table S1). Since noble gas concentrations in the residual gases increase due to the adsorption of acidic gases (such as CO_2_ and H_2_S) into the KOH solution in the bottles, those samples are excluded from the discussion about noble gas concentrations.

All measured ^3^He/^4^He ratios (2.5–8.1 R_A_) are significantly higher than the atmospheric value, indicating the presence of mantle He. The (^3^He/^4^He)_corr_ ratios (air-corrected ^3^He/^4^He) are calculated using the ^4^He/^20^Ne ratios (Eqs. 1 and 2). The (^3^He/^4^He)_corr_ ratios for Kitagawa fumaroles W (6.7–8.0 R_A_), C (6.2–8.1 R_A_), and E (7.1–8.0 R_A_) are generally higher than those for the Sesshogawara (6.4–7.8 R_A_) and Manza (5.3–7.4 R_A_) fumaroles (Supplementary Table S1). The (^3^He/^4^He)_corr_ ratio of the Motoshirane fumarolic gas (8.5 ± 2.1 R_A_) may be similar to those of the other fumaroles, although it has a large error due to the correction for significant atmospheric contamination. Because of the significant atmospheric contamination, the Motoshirane sample is excluded from the following discussions unless otherwise noted. According to Eq. 3, ^3^He_air_ contributes less than 9% of the sample ^3^He. Since the high (^3^He/^4^He)_corr_ ratios indicate crustal ^3^He is negligible, the ^3^He of most samples is dominantly derived from magma.

**Figure 2:**
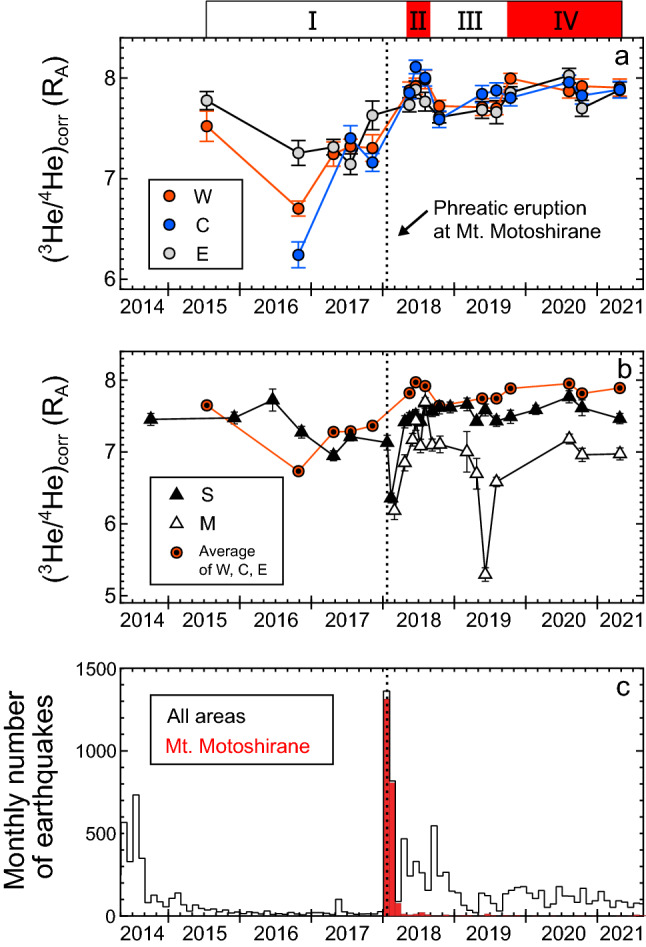
Temporal variations in air-corrected ^3^He/^4^He ((^3^He/^4^He)_corr_) ratios of fumarolic gases and monthly numbers of volcanic earthquakes at Kusatsu-Shirane volcano. (**a**) (^3^He/^4^He)_corr_ ratios measured at the W, C, and E fumaroles in the Kitagawa geothermal area. The Roman numerals above the panel are periods defined by the average (^3^He/^4^He)_corr_ ratios of the Kitagawa fumarolic gases. High average (^3^He/^4^He)_corr_ ratios (> 7.8 R_A_) were measured in periods II and IV. (**b**) (^3^He/^4^He)_corr_ ratios measured at the Sesshogawara (S) and Manza (M) fumaroles, and the averages of W, C, and E fumaroles. (**c**) Numbers of volcanic earthquakes per month in all areas of Kusatsu-Shirane (white) and near Mt. Motoshirane (red). The earthquake data are provided by the Japan Meteorological Agency. Dotted lines indicate 23 January 2018, when a phreatic eruption occurred at Mt. Motoshirane.

Figure 2 shows the temporal variations in the (^3^He/^4^He)_corr_ ratios for the Kitagawa, Sesshogawara, and Manza fumaroles, along with the monthly numbers of earthquakes at the Kusatsu-Shirane volcano. The dotted lines in Fig. 2a–c indicate a phreatic eruption that occurred at Mt. Motoshirane on 23 January 2018^29^. The temporal (^3^He/^4^He)_corr_ variation patterns for the three Kitagawa fumaroles W, C, and E are roughly synchronized. The (^3^He/^4^He)_corr_ ratios increased in May 2018, decreased in October 2018, and then progressively increased. We separated the study period since July 2015 into four intervals based on the averages of (^3^He/^4^He)_corr_ ratios for the three Kitagawa fumaroles (Fig. 2b). In periods I (July 2015–November 2017) and III (October 2018–August 2018), the average (^3^He/^4^He)_corr_ ratios were lower than the arbitrarily determined value of 7.8 R_A_. In periods II (May 2018–August 2018) and IV (October 2019–April 2021), the average (^3^He/^4^He)_corr_ ratios were higher than 7.8 R_A_. Period IV might have continued after the most recent sampling in April 2021.

The range of temporal variations for the Sesshogawara fumarole is smaller than that for the Kitagawa fumaroles except for the episodic drop in February 2018, three weeks after the 2018 eruption. The volcanic gases before the eruption were not sampled at the Manza fumarole. The (^3^He/^4^He)_corr_ ratios for the Manza fumarole progressively increased after the eruption until June 2018. In June 2019, the (^3^He/^4^He)_corr_ ratio episodically decreased and recovered in the next month.

### Temporal variations in carbon isotopic ratios and ^3^He/CO_2_ ratios

The δ^13^C-CO_2_ values for the Kitagawa fumaroles W, C, and E varied in the ranges of − 4.4 to − 1.4‰, − 4.9 to − 1.6‰, and − 4.2‰ to − 1.8‰, respectively. Those for the Sesshogawara and Manza fumaroles varied in the ranges of − 4.5 to − 0.8‰ and − 3.7 to − 0.5‰, respectively.

The ^3^He/CO_2_ ratios for the Kitagawa fumaroles W, C, and E significantly varied in the ranges of (0.3–1.6) × 10^−10^, (0.5–1.7) × 10^−10^, and (0.4–1.5) × 10^−10^, respectively. Those for the Sesshogawara and Manza fumaroles show relatively minor variations that are in the ranges of (0.3–0.7) × 10^−10^ and (0.3–0.5) × 10^−10^, respectively. The ^3^He/CO_2_ ratios of the W, C, and E samples collected on the same date are similar, and high in the periods II and IV when the (^3^He/^4^He)_corr_ ratios were high (Supplementary Fig. S2c).

**Figure 3:**
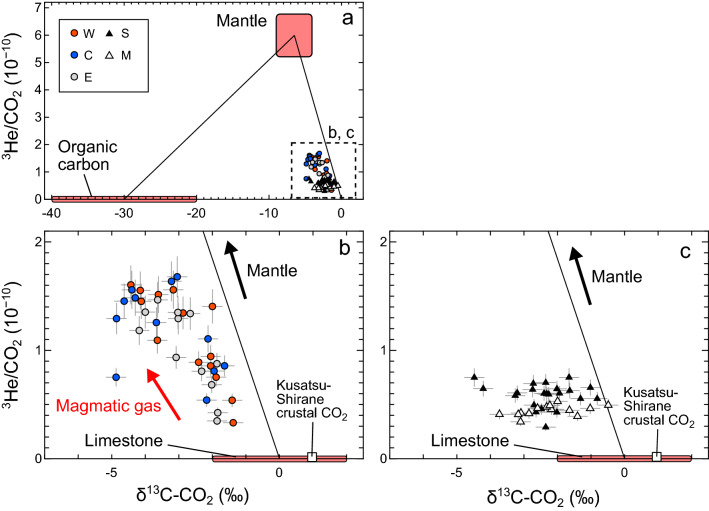
^3^He/CO_2_ versus δ^13^C-CO_2_ diagrams for the fumarolic gas samples. (**a**) Gas compositions for the W, C, and E fumaroles in the Kitagawa geothermal area and the Sesshogawara (S) and Manza (M) fumaroles. A dotted square indicates the areas of panels (**b**) and (**c**). (**b**) Gas compositions for the Kitagawa W, C, and E fumaroles. (**c**) Gas compositions for the S and M fumaroles. The mantle ^3^He/CO_2_ ratio of (5.99 ± 0.75) × 10^−10^ is after ref.^30^. The ranges of δ^13^C-CO_2_ values for mantle (− 4‰ to − 9‰), organic carbon (− 20‰ to − 40‰), and limestone (− 2‰ to 2‰) are after ref.^11^. The estimated δ^13^C-CO_2_ value (1.1‰) for Kusatsu-Shirane crustal CO_2_^10^ is also shown. Black lines are the mixing lines between mantle composition and organic-derived CO_2_, and mantle composition and limestone-derived CO_2_.

Figure 3 shows ^3^He/CO_2_ versus δ^13^C-CO_2_ diagrams. All data points are plotted within the region defined by the three-component mixing of the MORB-type mantle^11,30^, limestone-derived CO_2_^11^, and organic-derived CO_2_^11^ (Fig. 3). The ^3^He/CO_2_ ratios and the δ^13^C-CO_2_ values for the three Kitagawa fumaroles show a negative correlation (Fig. 3b). On the other hand, the ^3^He/CO_2_ ratios and the δ^13^C-CO_2_ values for the Sesshogawara and Manza fumaroles are poorly correlated (Fig. 3c).

### Temporal variations in ^3^He/^40^Ar* ratios

**Figure 4:**
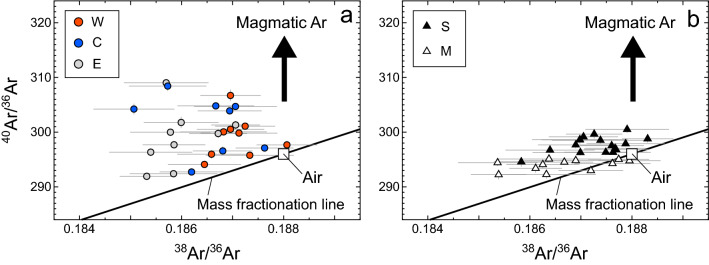
Ar three-isotope diagrams for the fumarolic gas samples collected at the W, C, and E fumaroles in the Kitagawa geothermal area, and the Sesshogawara (S) and Manza (M) fumaroles. The air composition of (^38^Ar/^36^Ar, ^40^Ar/^36^Ar) = (0.188, 296) and a mass fractionation line for the atmospheric Ar are also shown^3^.

Figure 4 shows the ^40^Ar/^36^Ar versus ^38^Ar/^36^Ar diagrams. The black line is a mass fractionation line for the atmospheric Ar^3^. Most data points are plotted above the atmospheric mass fractionation line, indicating that those fumarolic gases contain excess ^40^Ar (i.e., ^40^Ar*), that may originate in the mantle or crust where radiogenic ^40^Ar has been produced by the decay of ^40^ K. The sample ^38^Ar/^36^Ar ratios show negative anomalies up to ~ 1.6% relative to the air. The anomalies reflect mass fractionation because the mantle ^38^Ar/^36^Ar ratio is almost indistinguishable from the atmospheric value^3^. Because such isotopic shifts should be parallel to the atmospheric mass fractionation line, the ^40^Ar* concentrations are calculated from the vertical deviations from the atmospheric mass fractionation line using Eqs. 4 and 5. Some samples have large ^40^Ar* errors or no resolvable ^40^Ar excess due to significant contributions of atmospheric Ar.

The ^40^Ar* concentrations in the Kitagawa samples are higher than ~ 0.8 ppm (Supplementary Table S1). Assuming that the crustal ^4^He/^40^Ar ratio is ~ 6 (a typical production ratio in the crust^5^), the crustal ^40^Ar concentrations in the samples are estimated from the crustal ^4^He concentrations. Assuming that the (^3^He/^4^He)_corr_ ratios reflect a simple two-component mixing of the crustal and magmatic He with ^3^He/^4^He ratios of 0.02 R_A_^4^ and 8.1 R_A_ (the highest (^3^He/^4^He)_corr_ ratio in this study), respectively, crustal ^4^He concentrations in all the samples are estimated. Accordingly, the crustal ^40^Ar in the samples should be less than 9% of their total ^40^Ar*. Therefore, the most ^40^Ar* of the Kitagawa samples are derived from the magma containing mantle Ar. Some Sesshogawara and Manza samples also contain detectable ^40^Ar*. The crustal ^40^Ar in these samples contributes less than 7% of their total ^40^Ar*.

In summary, both ^3^He and ^40^Ar* in the samples are mostly magmatic in origin. Because of their inertness, the ^3^He/^40^Ar* variations of the fumarolic gases may directly reflect the ^3^He/^40^Ar* variations of magmatic gas. Some ^3^He/^40^Ar* ratios have large errors due to the large uncertainties of ^40^Ar*. In order to examine the ^3^He/^40^Ar* variation properly, we used the ^3^He/^40^Ar* ratios with < 70% errors in the following discussion. These are 35 of the 63 samples for which Ar isotopic compositions were measured.

**Figure 5:**
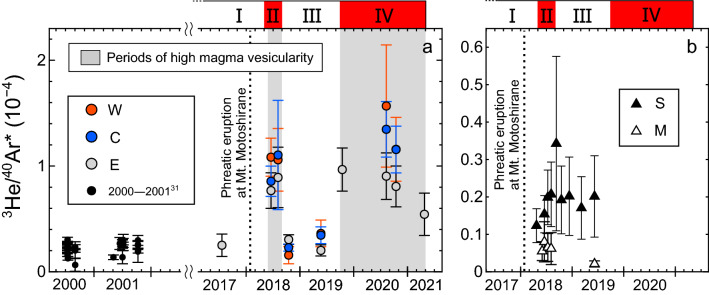
Temporal variations in the ^3^He/^40^Ar* ratios of fumarolic gases. (**a**) ^3^He/^40^Ar* ratios measured at the W, C, and E fumaroles in the Kitagawa geothermal area and those of fumarolic gases collected in the same area in 2000–2001^31^. Gray areas highlight the periods of high magma vesicularity, which are suggested by increased ^3^He/^40^Ar* ratios. (**b**) ^3^He/^40^Ar* ratios measured at the Sesshogawara (S) and Manza (M) fumaroles. The Roman numerals above the panels are periods defined by the average (^3^He/^4^He)_corr_ ratios of the Kitagawa fumarolic gases (see text for details). Samples with uncertainties of more than 70% in the ^3^He/^40^Ar* ratios are not shown. Dotted lines indicate 23 January 2018, when a phreatic eruption occurred at Mt. Motoshirane.

Figure 5 shows temporal variations in the ^3^He/^40^Ar* ratios for the Kitagawa, Sesshogawara, and Manza fumaroles along with the ^3^He/^40^Ar* ratios of the fumarolic gases collected at the Kitagawa geothermal area in 2000–2001 ((0.1–0.3) × 10^−4^; calculated from the literature data^31^). The ^3^He/^40^Ar* ratios for the three Kitagawa fumaroles synchronously varied in the range of (0.2–1) × 10^−4^, and the ^3^He/^40^Ar* ratios were high during the high (^3^He/^4^He)_corr_ periods II and IV (Fig. 5). The ^3^He/^40^Ar* ratios for the Sesshogawara fumarole were almost stable in the range of (0.1–0.3) × 10^−4^. The ^3^He/^40^Ar* ratios for the Manza fumarole were (0.02–0.1) × 10^−4^.

## Discussion

As previously mentioned, the (^3^He/^4^He)_corr_ ratios of the fumarolic gases reflect the mixing of magmatic and crustal He. The synchronous temporal variations in the (^3^He/^4^He)_corr_ ratios for the three Kitagawa fumaroles suggest that they are fed by a common volcanic gas reservoir. The highest observed (^3^He/^4^He)_corr_ ratio of 8.1 R_A_ indicates that the ^3^He/^4^He ratio of the Kusatsu-Shirane magma is ~ 8.1 R_A_ or higher. This is in the range of the ^3^He/^4^He ratios of mid-ocean ridge basalts (7–9 R_A_^4^). Therefore, the Kusatsu-Shirane magma is almost unaffected by crustal He. The (^3^He/^4^He)_corr_ ratios for the Sesshogawara and Manza fumaroles are lower than those for the Kitagawa fumaroles, reflecting a greater influence of crustal He.

The average (^3^He/^4^He)_corr_ ratio of the Kitagawa fumarolic gases since May 2018 (~ 7.8 R_A_) is higher than that before November 2017 (~ 7.2 R_A_). The increase in (^3^He/^4^He)_corr_ ratios can be explained by an increase in magmatic He supply or a decrease in crustal He supply. For the Kitagawa fumaroles, the former is more likely because this is consistent with the enrichments in magmatic gas species (such as CO_2_ and He) in the fumarolic gases since May 2018^28^. Thus, the magmatic gas supply may have become more substantial since sometime between November 2017 and May 2018. This view is supported by previous reports of shallow inflation and more volcanic earthquakes around Mt. Shirane since April 2018, three months after the 2018 eruption^21,25^ (Fig. 2c). The enhanced volcanic activity may reflect the supply of magmatic fluid from a magma chamber, a potential pressure source for deep inflation at a depth of ~ 4 km since 2018^21,26^.

It should be noted that some previous studies proposed a mixing of two magmatic components with different ^3^He/^4^He ratios to explain (^3^He/^4^He)_corr_ variations of volcanic gases^15,32–34^. In this case, one component with a higher ^3^He/^4^He ratio may originate from a primitive magma. The other component with a lower ^3^He/^4^He ratio may originate from an aged magma, which might have experienced crustal contamination and magma aging that can lower the ^3^He/^4^He ratio^15^. However, this may not be the case for the Kusatsu-Shirane volcano, where the ^3^He/^40^Ar* and ^3^He/CO_2_ ratios are low when the (^3^He/^4^He)_corr_ ratio is low (Fig. 5 and supplementary Fig. S2c). This relationship is opposite to the expected composition of aged (more degassed) magma, which should have higher ^3^He/^40^Ar* and ^3^He/CO_2_ ratios because the solubilities of Ar and CO_2_ in silicate melt are lower than He^35,36^.

The magmatic gas supply might have been especially large during periods II (May 2018–August 2018) and IV (October 2019–April 2021) when relatively high (^3^He/^4^He)_corr_ ratios are observed. This is supported by the recent variation in the Cl concentration of the Yugama crater lake, suggesting an increase in the supply of magmatic fluid in 2018 and 2020^27,37^. This result confirms that the (^3^He/^4^He)_corr_ ratio is an excellent parameter to monitor the temporal variations in magmatic gas supply at a volcano with a well-developed hydrothermal system that may interfere with the compositions of other chemically reactive magmatic gas species, as has been previously proposed by the studies at the other volcanoes^6–8^.

At the Sesshogawara and Manza fumaroles, significantly low (^3^He/^4^He)_corr_ ratios were measured after the phreatic eruption in 2018 (Fig. 2), suggesting a decrease in the magmatic/crustal He ratios in response to the eruption. The synchronous responses may reflect that both fumaroles are connected to the hydrothermal fluid reservoir that caused the phreatic eruption at Mt. Motoshirane^21^. This is consistent with a magnetotelluric study^23^ that suggested the presence of a hydrothermal fluid reservoir (the C2 conductor in Fig. 1) broadly spread beneath the Kusatsu-Shirane volcano.

During the 2018 eruption, fluid injection from the hydrothermal fluid reservoir to shallower depths was suggested from ground deformation recorded by a borehole tiltmeter network at the Kusatsu-Shirane volcano^21^. The low (^3^He/^4^He)_corr_ ratios after the eruption might indicate that the hydrothermal fluid in the deeper part of the fluid reservoir is more influenced by crustal He than the shallower part, which constantly supplies fluid to the fumaroles. In this case, the fluid with a low ^3^He/^4^He ratio might have been temporarily supplied from the deeper part to the fumaroles. Another process that would lower the (^3^He/^4^He)_corr_ ratios is a decrease in the hydrothermal fluid supply to the fumaroles. A previous study reported a gradual decrease in the (^3^He/^4^He)_corr_ ratios of fumarolic and hot spring gases with distance from the center of volcanic activity at the Kusatsu-Shirane volcano (i.e., the Yugama crater lake), indicating the continuous addition of crustal He during the transport of hydrothermal fluid^10^. Since the hydrothermal fluid is the carrier of magmatic He with a high ^3^He/^4^He ratio, the ^3^He/^4^He ratios of fumarolic gases would become lower when the fluid supply decreases.

The low (^3^He/^4^He)_corr_ ratio was measured at the Manza fumarole alone in June 2019. In this case, the scenario of low-^3^He/^4^He fluid injection is less likely because it would also result in a low (^3^He/^4^He)_corr_ ratio at the Sesshogawara fumarole. Therefore, the low (^3^He/^4^He)_corr_ ratio in June 2019 suggests a decrease in the hydrothermal fluid supply to the Manza fumarole.

Some physicochemical reactions such as (1) vapor–liquid separation, (2) pH-related CO_2_-HCO_3_^−^ equilibrium, and (3) carbonate precipitation can modify ^3^He/CO_2_ and δ^13^C-CO_2_ values of volcanic gases at a magmatic‒hydrothermal system^2,10,38,39^. For our samples, (2) and (3) are ruled out because both require medium to high-pH conditions, although the magmatic‒hydrothermal fluid of the Kusatsu-Shirane volcano is strongly acidic (pH < 3.2)^10,27,40^. The (1) vapor–liquid separation may cause the ^3^He/CO_2_ fractionation due to the lower solubility of He than CO_2_ in aqueous fluid^41^. However, because the bulk of the volatiles partition into the gas phase, the ^3^He/CO_2_ ratios of the sampled gas phase are expected to approach that of the fluid before vapor–liquid separation^2^. Accordingly, the ^3^He/CO_2_ and δ^13^C-CO_2_ variations may not be attributable to the fractionation due to the above reactions.

The Kitagawa fumarolic gas compositions show a negative correlation in the ^3^He/CO_2_ versus δ^13^C-CO_2_ diagram, and the data points with lower ^3^He/CO_2_ ratios distribute toward the limestone-derived CO_2_ composition (Fig. 3b). A previous study reported that δ^13^C-CO_2_ values of volcanic gases from the Kusatsu-Shirane volcano increase with distance from the Yugama crater, suggesting the shallow assimilation of crustal CO_2_ produced by decarbonation of the limestones with a δ^13^C-CO_2_ value of 1.1‰ around the Kusatsu-Shirane volcano^10^. Therefore, the negative correlations most likely reflect the mixing of the magmatic gas and the crustal CO_2_ with various proportions (Fig. 3b). This view is supported by the fact that the high ^3^He/CO_2_ ratios were observed in the periods II and IV when the (^3^He/^4^He)_corr_ ratios were high, reflecting large magmatic gas contribution (Supplementary Fig. S2). Note that the ^3^He/CO_2_ and δ^13^C-CO_2_ values of the magmatic gas are probably fractionated during magmatic degassing as discussed later.

For the Sesshogawara and Manza fumaroles, the lower ^3^He/CO_2_ ratios than the Kitagawa fumaroles suggest smaller magmatic CO_2_ contributions. This is consistent with the lower (^3^He/^4^He)_corr_ ratios for both fumaroles (Fig. 2), indicating smaller contributions of magmatic He. The δ^13^C-CO_2_ variations of more than a few ‰, independent of the ^3^He/CO_2_ ratios, may reflect the addition of various amounts of organic-derived CO_2_ at shallow depths. The soil CO_2_ from some parts of the Satsuma-Iwojima volcano mainly consists of biogenic CO_2_ with a low δ^13^C-CO_2_ of − 27‰^42^. Similar soil CO_2_ dissolved in meteoric water is a potential source of the organic-derived CO_2_ since the incorporation of meteoric water has been suggested from the elemental and isotopic compositions of the Sesshogawara and Manza fumarolic gases^28^.

The fractionation of ^3^He/^40^Ar* ratios in the gas phase during vapor–liquid separation should be minimal for our samples because the solubilities of He and Ar in water are similarly low, and the difference is much smaller than those of He and CO_2_^5,41^. Therefore, the ^3^He/^40^Ar* ratios in the fumarolic gases are expected to be almost identical to the magmatic gas composition. The ^3^He/^40^Ar* ratios of the Kitagawa fumarolic gases have episodically increased since May 2018. The highest ratios were ~ 5 times the pre-eruptive value of ~ 3 × 10^−5^ in 2017 (Fig. 5). In addition, the ^3^He/^40^Ar* ratios of (1–3) × 10^−5^ in 2000–2001^31^ were similar to the pre-eruptive value. Since the volcano had been calm between 1991 and 2014^43,44^, these values indicate that the ^3^He/^40^Ar* ratios were low during low volcanic activity.

**Figure 6:**
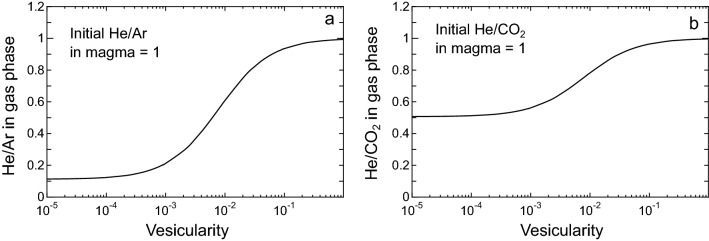
He/Ar and He/CO_2_ fractionations in the gas phase during closed system degassing of basaltic melt. Black lines show the fractionation lines computed using Eq. 6 with assumptions that the solubilities of He, Ar, and CO_2_ in the melt are 5.7 × 10^−4^ cm^3^STP g^−1^ bar^−1^, 6.4 × 10^−5^ cm^3^STP g^−1^ bar^−1^, and 2.9 × 10^−4^ cm^3^STP g^−1^ bar^−1^, respectively^35,36^. The initial He/Ar and He/CO_2_ ratios in magma are 1. See “Methods” section for details.

**Figure 7:**
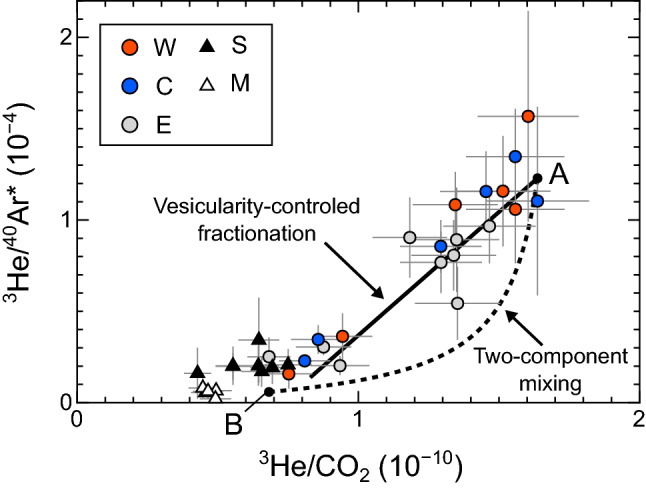
^3^He/^40^Ar* versus ^3^He/CO_2_ diagram for the fumarolic gas samples. Samples with uncertainties of more than 70% in the ^3^He/^40^Ar* ratios are not shown. The straight line shows a relationship between ^3^He/^40^Ar* and ^3^He/CO_2_ ratios in the gas phase of a magma with a composition of “A” as a function of magma vesicularity. The relationship is computed using Eq. 6 with assumptions that the solubilities of He, Ar, and CO_2_ in the magma are 5.7 × 10^−4^ cm^3^STP g^−1^ bar^−1^, 6.4 × 10^−5^ cm^3^STP g^−1^ bar^−1^, and 2.9 × 10^−4^ cm^3^STP g^−1^ bar^−1^, respectively^35,36^. The dashed curve represents a two-component mixing curve for gases with compositions of “A” and “B”. The points “A” and “B” are on the linear regression line for the Kitagawa fumarolic samples with the maximum (1.7 × 10^−10^) and minimum (6.8 × 10^−11^) ^3^He/CO_2_ ratios, respectively.

Volatiles in a gas phase separated from a silicate melt are fractionated as functions of solubilities and melt vesicularity. Volatiles with lower solubilities are more partitioned into the gas phase, and the degree of elemental fractionation decreases with increasing vesicularity^1,45^. Figure 6 shows the fractionations of He/Ar and He/CO_2_ ratios in the gas phase of an anhydrous basaltic magma as functions of magma vesicularity in a closed system (computed from Eq. (6) in “Methods” section). Since the solubility of Ar in a silicate melt is lower than He^35^, the He/Ar ratio in the gas phase is low when the magma vesicularity is small. Although the solubility difference between He and Ar may decrease when the H_2_O concentration in a silicate melt is higher, the solubility of He is always higher than Ar^46^. Therefore, the increase in ^3^He/^40^Ar* ratios is attributable to the enhanced vesicularity of the degassing magma. This inference is supported by the compositional trend for Kitagawa samples showing a linear correlation in the ^3^He/^40^Ar* versus ^3^He/CO_2_ diagram (Fig. 7) because a vesicularity-controlled fractionation trend is expected to be linear. For example, the vesicularity-controlled fractionation trend for the gas phase of a magma with a composition of “A”, calculated from Eq. 6 and the parameters used for Fig. 6, yields the straight line in Fig. 7. The enhanced magma vesicularity is consistent with an increase in the magmatic gas supply since 2018, inferred from the (^3^He/^4^He)_corr_ ratios. Another process that would potentially change the ^3^He/^40^Ar* ratios is a mixing of two (or more) magmatic components with different compositions. However, this is less likely because two-component mixing yields a curved correlation in the ^3^He/^40^Ar* versus ^3^He/CO_2_ diagram, as demonstrated by the mixing curve between “A” and “B” in Fig. 7, which is different from the trend for the Kitagawa samples.

The linear correlation also indicates that the ^3^He/CO_2_ variations in the Kitagawa fumarolic gases during the periods of high ^3^He/CO_2_ ratios (> ~ 1 × 10^−10^) mainly reflect vesicularity-controlled fractionation, suggesting that the CO_2_ is mostly magmatic during these periods. The highest ^3^He/CO_2_ ratio (~ 1.7 × 10^−10^) is much lower than the MORB-type mantle value ~ 6 × 10^−10^ (ref.^30^) despite the most ^3^He is also magmatic in origin. The low ^3^He/CO_2_ ratios support the previous study that suggested the ^3^He/CO_2_ ratio of the Kusatsu-Shirane magma is lower than the MORB-type mantle value due to the addition of CO_2_ derived from subducted materials^11^. The δ^13^C-CO_2_ values of the Kitagawa samples with high ^3^He/CO_2_ ratios (> ~ 1 × 10^−10^) have a wide range from − 4.9 to − 2.0‰. It is known that the δ^13^C-CO_2_ exsolved from the magma is fractionated to a few ‰ heavier value than that in the melt^47^. The δ^13^C-CO_2_ variation may reflect a complex fractionation during magma degassing. However, it cannot be excluded that the variation is due to the assimilation of organic-derived CO_2_ at shallower depths like that proposed for the Sesshogawara and Manza fumaroles.

**Figure 8:**
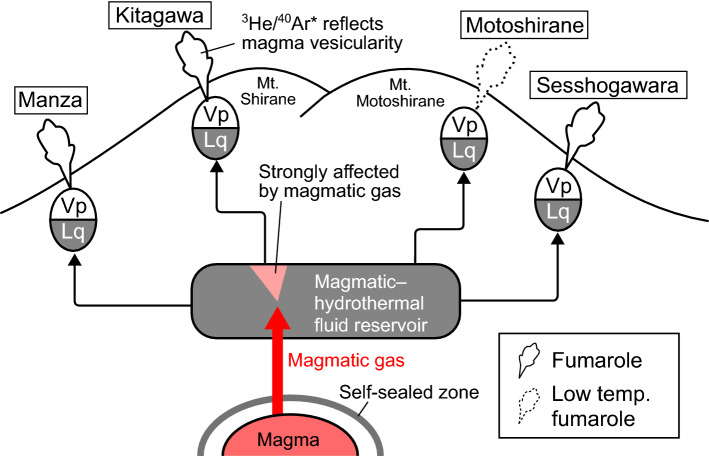
Schematic illustration for the magmatic-hydrothermal system at the Kusatsu-Shirane volcano. Black arrows indicate fluid supplies from a magmatic–hydrothermal fluid reservoir. Abbreviations: Vp, vapor phase; Lq, liquid phase.

Noble gases are minor volatile species in magma and do not affect vesiculation. Instead, the enhancement in magma vesicularity occurs when the magma is depressurized or the concentrations of major volatile species in the magma (e.g., H_2_O and CO_2_) increase. The depressurization of magma may be caused by magma ascent or breakdown of a self-sealed zone (Fig. 8), which is mainly formed by the precipitation of silica at the brittle‒ductile transition zone (370–400 °C) around the magma and may induce overpressure^27,28,48^. On the other hand, the increase in the volatile concentration may be caused by an addition of volatile-rich magma or a decrease in the melt volume as a result of crystallization. However, the former process is less likely because the long-term decreasing trend of the SO_4_/Cl ratio of the Yugama crater lake since early 2000s is inconsistent with an intrusion of new magma that may cause an increase in the SO_4_/Cl ratio^27^. The latter process is inconsistent with the rapid enhancement in magma vesicularity because crystallization is a continuous process during the entire magma cooling history^18^. Therefore, depressurization due to magma ascent or breakdown of a self-sealed zone might have caused the enhancement in magma vesicularity in periods II and IV.

Here, we summarize a possible scenario for the temporal variations in the magmatic gas composition at the Kusatsu-Shirane volcano since 2014. The enhancement in magma vesicularity started in April 2018 and increased the ^3^He/^40^Ar* ratio of the gas exsolved from the magma. Then, magmatic gas with a high ^3^He/^40^Ar* ratio was supplied to the Kitagawa fumaroles, resulting in the episodic increases in the ^3^He/^40^Ar* ratios observed in period II (Fig. 5a). The enhanced magma vesicularity also increased the magma gas supply, resulting in high (^3^He/^4^He)_corr_ ratios (Fig. 2a). The low ^3^He/^40^Ar* ratios in period III suggest a short-term decrease in magma vesicularity, possibly due to pressurization by magma descent or growth of a self-sealed zone^27,28,48^. The high ^3^He/^40^Ar* ratios in period IV indicate that the magma vesicularity increased again.

Figure 8 shows a schematic illustration of the magmatic–hydrothermal system at the Kusatsu-Shirane volcano that summarizes the discussion above. The hydrothermal fluid reservoir broadly extending beneath the Kusatsu-Shirane volcano^23^ may supply magmatic–hydrothermal fluid to the fumaroles around the volcano. The fluid is heterogeneously influenced by magmatic gas. The composition of fluid feeding the Kitagawa fumarolic gases is significantly affected by the magmatic gas, and the ^3^He/^40^Ar* variation due to the changes in magma vesicularity is detectable. The fluid compositions feeding the Sesshogawara fumarole and the Manza fumarole are also affected by the magmatic gas, but not sensitive to the temporal variations of magmatic gas composition.

## Conclusion

We measured the concentrations and isotopic compositions of noble gases and CO_2_ in fumarolic gases collected repeatedly at the Kusatsu-Shirane volcano between October 2014 and April 2021. The variations in air-corrected ^3^He/^4^He ratios suggest that the magmatic gas supply increased a few months after the phreatic eruption at Mt. Motoshirane in January 2018, then decreased between October 2018 and August 2019, and then increased again after October 2019. The relationship between the ^3^He/CO_2_ ratios and δ^13^C-CO_2_ values also supports the temporal variations in magmatic gas contribution. Significant increases in ^3^He/^40^Ar* ratios were detected in the periods of large magmatic gas supply. The increase probably reflects enhancement in the magma vesicularity. The detection of the ^3^He/^40^Ar* variation strongly supports that the ^3^He/^40^Ar* ratio is useful to monitor the current state of degassing magma, which is essential for understanding the deep process of volcanic unrest and may contribute to identifying precursors of a future eruption. Since fumaroles are commonly observed at active volcanoes, the monitoring of a magmatic–hydrothermal system by noble gas and carbon isotopic compositions of fumarolic gases should be applicable to other volcanoes.

## Methods

### Gas sampling

Fumarolic gas samples were collected at six sites at the Kusatsu-Shirane volcano (Fig. 1). Three sites (W, C, E) are in the Kitagawa geothermal area located on the northern side of Mt. Shirane. The locations of W, C, E, Sesshogawara (S), and Manza (M) are the same as those of W, C, E, S, and M in ref.^28^, respectively. The temperatures of fumarolic gases were almost at the boiling points of water at the elevations of the sampling sites during the sampling period, except for the Motoshirane fumarolic gas. The temperature of the Motoshirane fumarolic gas was almost the same as the ambient temperature, and the gas was not accompanied by wet steam.

In most cases, the gas samples were collected in 50 ml glass sample containers with vacuum valves at both ends. The fumarolic gas was introduced into the container from a fumarolic vent using a titanium pipe and Tygon tubes through a 100 ml glass bottle with vacuum valves at both ends. The 100 ml glass bottle was cooled in ice water to condense the water vapor of the fumarolic gas. The “dry” gas sample from which water vapor was almost completely removed by condensation was collected after flushing the sample container several times with fumarolic gas. The pH values of the condensed water were lower than 4.4 for all samples, indicating that CO_2_ dissolution into the condensed water was negligible. Therefore, the δ^13^C values of CO_2_ in the “dry” gas samples were assumed to be the same as those in the fumarolic gases.

Some samples were collected in 120 ml Giggenbach-type glass bottles containing 5 molar 20 ml KOH solution, following the method described in ref.^28^. Since water and acidic gases are adsorbed into the KOH solution, noble gases reside in the headspace of the bottle as a residual gas (R-gas). The samples collected in the Giggenbach-type bottles are tagged as “yes” in the R-gas column of Supplementary Table S1.

### Noble gas measurements

Noble gas analysis was carried out using a noble gas mass spectrometer (modified VG5400/MS-IV) at the University of Tokyo. Details on the mass spectrometric system and basic analytical procedure are the same as those described in refs.^49,50^. Air standards were frequently measured during the analysis to determine the mass discrimination factors and the sensitivities of the mass spectrometers for all noble gases except for helium isotopic ratios. The correction factor for the helium isotopic ratios was determined using an interlaboratory helium standard named HESJ. The recommended ^3^He/^4^He ratio of HESJ is 20.63 ± 0.10 R_A_^51^. The errors in the noble gas isotopic ratios are 1 SD, including statistical errors during sample analysis, errors in the discrimination factors, and the error in the He standard gas. Uncertainties of the concentrations are assumed to be 5% for He, Ne, and Ar and 10% for Kr and Xe, based on the reproducibility of standard gas measurements.

### CO_2_ measurements

After noble gas analysis, the ^13^C/^12^C ratios (expressed as δ^13^C_PDB_ values) and concentrations of CO_2_ in the gas samples were measured using a gas chromatography, combustion and mass spectrometry (GC/C/MS) system (Delta-S, Finnigan MAT instrument) at the University of Tokyo. The mass discrimination for δ^13^C and the sensitivity for CO_2_ of the GC/C/MS system were calibrated by standard gas measurements (CO_2_ > 99.95%, δ^13^C =  − 30.90‰), which were intermittently carried out during sample analysis. Uncertainties of the CO_2_ concentrations and the δ^13^C-CO_2_ values are assumed to be 10% and 0.3‰, respectively, based on the reproducibility of standard gas measurements.

### Calculation of (^3^He/^4^He)_corr_ ratios

The (^3^He/^4^He)_corr_ ratios (air-corrected ^3^He/^4^He) are calculated by the following equations assuming that the ^4^He/^20^Ne ratios of magmatic and crustal components are significantly higher than that of air^52^:

1 $$\left( {{}_{{}}^{3} {\text{He}}/{}_{{}}^{4} {\text{He}}} \right)_{{{\text{corr}}}} = \frac{{\left( {{}_{{}}^{3} {\text{He}}/{}_{{}}^{4} {\text{He}}} \right)_{{{\text{sample}}}} - r}}{1 - r}$$

2 $$r = \frac{{\left( {{}_{{}}^{4} {\text{He}}/{}_{{}}^{20} {\text{Ne}}} \right)_{{{\text{air}}}} }}{{\left( {{}_{{}}^{4} {\text{He}}/{}_{{}}^{20} {\text{Ne}}} \right)_{{{\text{sample}}}} }}$$

where (^4^He/^20^Ne)_air_ of 0.318 is assumed^3^.

### Calculation of ^3^He_air_/^3^He_sample_ ratios

The fractions of ^3^He_air_ in the samples are calculated by the following equation assuming that all ^20^Ne is derived from the air:

3 $$\frac{{{}_{{}}^{3} {\text{He}}_{{{\text{air}}}} }}{{{}_{{}}^{3} {\text{He}}_{{{\text{sample}}}} }}{ } = \frac{{\left( {{}_{{}}^{20} {\text{Ne}}/{}_{{}}^{4} {\text{He}}} \right)_{{{\text{sample}}}} /\left( {{}_{{}}^{3} {\text{He}}/{}_{{}}^{4} {\text{He}}} \right)_{{{\text{sample}}}} }}{{\left( {{}_{{}}^{20} {\text{Ne}}/{}_{{}}^{4} {\text{He}}} \right)_{{{\text{air}}}} /\left( {{}_{{}}^{3} {\text{He}}/{}_{{}}^{4} {\text{He}}} \right)_{{{\text{air}}}} }}$$

### Calculation of ^40^Ar* concentrations

The concentration of excess ^40^Ar (= ^40^Ar*) is given by:

4 $$^{{{4}0}} {\text{Ar}}* \, = \, [\left( {^{{{4}0}} {\text{Ar}}/^{{{36}}} {\text{Ar}}} \right) - \left( {^{{{4}0}} {\text{Ar}}/^{{{36}}} {\text{Ar}}} \right)_{{{\text{atm}}}} ] \, \times^{{{36}}} {\text{Ar}}$$

where (^40^Ar/^36^Ar)_atm_ is the ^40^Ar/^36^Ar ratio of atmospheric Ar in a sample. The (^40^Ar/^36^Ar)_atm_ ratio is given by:

5 $$\left( {^{{{4}0}} {\text{Ar}}/^{{{36}}} {\text{Ar}}} \right)_{{{\text{atm}}}} = { 3}0{31 } \times \, \left( {^{{{38}}} {\text{Ar}}/^{{{36}}} {\text{Ar}}} \right) - {273}.{8}$$

where 3031 and − 273.8 are the slope and intercept of the mass fractionation line for atmospheric Ar, respectively. The mass fractionation line is calculated assuming the Rayleigh process and the air Ar values of (^38^Ar/^36^Ar, ^40^Ar/^36^Ar) = (0.188, 296)^3^.

### Fractionation of He/Ar and He/CO_2_ ratios in the gas phase of magma

Since growing bubbles in a silicate melt quickly reach and maintain chemical equilibrium with the surrounding liquid, nonequilibrium exsolution might be due only to dramatic depressurization occurring during explosive volcanic activity^18^. Therefore, the ^3^He/^40^Ar* variations were not likely to be produced by kinetic fractionation caused by the difference in He and Ar diffusivities.

The noble gases in a gas phase exsolved from a silicate melt are fractionated as functions of solubilities and melt vesicularity^45^:

6 $$\left( {\frac{{C_{i} }}{{C_{j} }}} \right)_{{{\text{gas}}}} = \left( {\frac{{C_{i} }}{{C_{j} }}} \right)_{0} \times \frac{{V^{*} + \rho S_{j} T_{e} /T_{0} }}{{V^{*} + \rho S_{i} T_{e} /T_{0} }}$$

where (*C*_*i*_/*C*_*j*_)_gas_ is the abundance ratio of species *i* and *j* in the gas phase, (*C*_*i*_/*C*_*j*_)_0_ is the initial abundance ratio in the melt, V^*^ is the vesicularity of the melt of density *ρ, S*_*i*_ and *S*_*j*_ are the solubilities, *T*_*e*_ is the equilibrium temperature, and *T*_*0*_ is 273 K.

According to Eq. 6, noble gases with lower solubilities are preferentially partitioned into the gas phase, and the degree of fractionation decreases with increasing vesicularity. The solubility of Ar in a silicate melt is lower than that of He by a factor of 6–11 (ref.^35^). Therefore, the increases in ^3^He/^40^Ar* ratios are attributable to the increased vesicularity of degassing magma. The fractionation of He/Ar in a gas phase of basaltic magma is computed by using Eq. 6 with assumptions that the solubilities for He and Ar are 5.7 × 10^−4^ cm^3^STP g^−1^ bar^−1^ and 6.4 × 10^−5^ cm^3^STP g^−1^ bar^−1^ (alkali olivine basalt at 1350°C^35^), respectively, *ρ* is 2.8 g cm^−3^, and T_C_ is 1350 °C (Fig. 6a). Since the solubility of Ar is ~ 10 times lower than that of He, the He/Ar ratio in the gas phase can vary by a factor of ~ 10 at a maximum. Similarly, the fractionation of He/CO_2_ is also computed by assuming a CO_2_ solubility of 2.9 × 10^−4^ cm^3^STP g^−1^ bar^−1^ (0.567 ppm bar^−1^ in anhydrous silicate melts, ref.^36^) (Fig. 6b).

## Supplementary Information

Supplementary Information 1.

Supplementary Information 2.

## Data Availability

All data obtained in this study are included in the Supplementary Data file.
