# Supplementary material for: Monitoring of magmatic–hydrothermal system by noble gas and carbon isotopic compositions of fumarolic gases
Source: Sci Rep. 2022 Nov 21;12:17967. doi: 10.1038/s41598-022-22280-3 (PMC9678900; doi:10.1038/s41598-022-22280-3)
Supplement: Supplementary file 1 — Supplementary Information 1. [file 41598_2022_22280_MOESM1_ESM.pdf]

Supplementary Materials for

Monitoring of magmatic–hydrothermal system by noble gas and carbon isotopic compositions of fumarolic gases

Tomoya Obase [1,2]*, Hirochika Sumino [1,3], Kotaro Toyama [1,4], Kaori Kawana [1,5], Kohei Yamane [1], Muga Yaguchi [6], Akihiko Terada [7], and Takeshi Ohba [8]

[1] Department of General Systems Studies, Graduate School of Arts and Sciences, The University of Tokyo, 3-8-1 Komaba, Meguro, Tokyo 153-0041, Japan.
[2] Department of Earth and Planetary Sciences, Faculty of Science, Hokkaido University, Sapporo, Hokkaido 060-0810, Japan.
[3] Research Center for Advanced Science and Technology, The University of Tokyo, 4-6-1 Komaba, Meguro-ku, Tokyo 153-0041, Japan
[4] Hot Springs Research Institute of Kanagawa Prefecture, Odawara, Kanagawa 250-0031, Japan.
[5] Earth Surface System Research Center, Research Institute for Global Change, Japan Agency for Marine-Earth Science and Technology (JAMSTEC), Yokohama, Kanagawa 236-0001, Japan.
[6] Meteorological Research Institute, Japan Meteorological Agency, 1-1 Nagamine, Tsukuba, Ibaraki 305-0052, Japan.
[7] Volcanic Fluid Research Center, School of Science, Tokyo Institute of Technology, 2-12-1 Ookayama, Meguro-ku, Tokyo 152-8551, Japan.
[8] Department of Chemistry, School of Science, Tokai University, 4-1-1 Kitakaname, Hiratsuka, Kanagawa 259-1291, Japan.

*Corresponding author: Tomoya Obase
Department of Earth and Planetary Sciences, Faculty of Science, Hokkaido University, Sapporo, Hokkaido 060-0810, Japan.
E-mail: obase@sci.hokudai.ac.jp

This PDF file includes the following sections:
Supplementary Figs. S1 and S2

Supplementary Figures

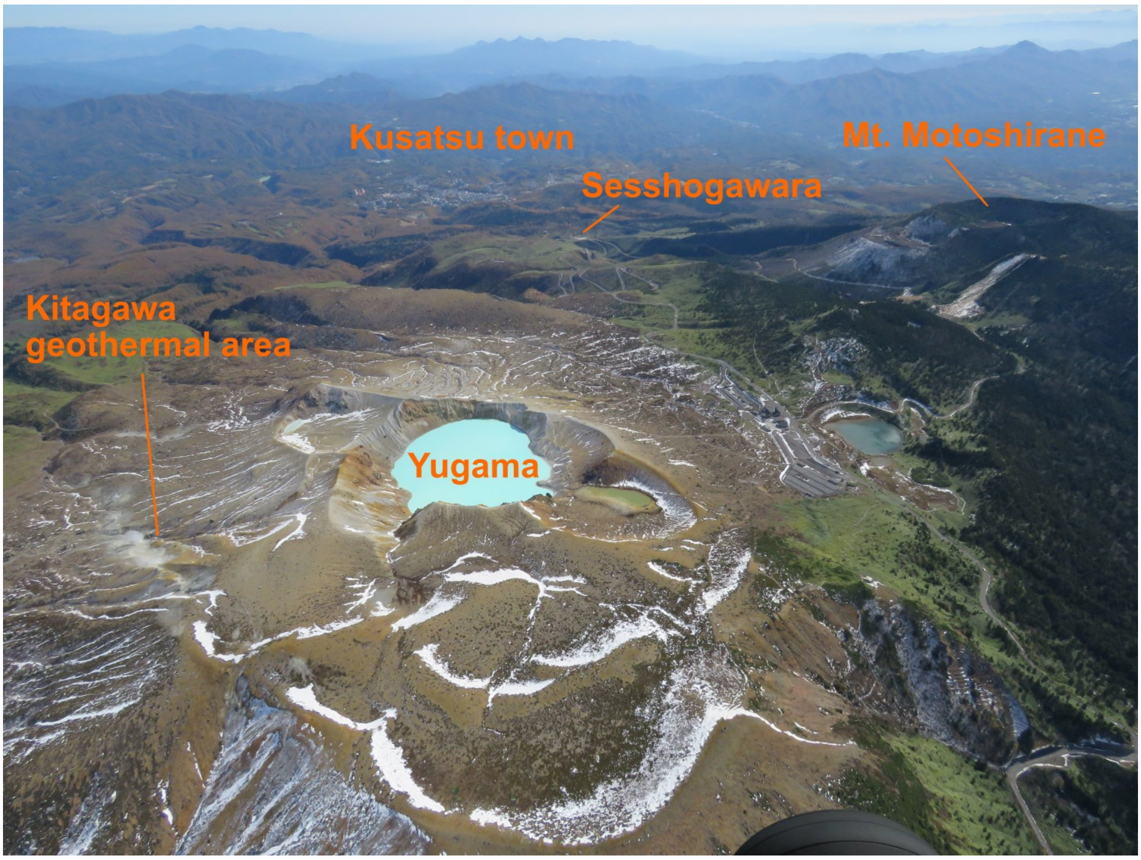

Fig. S1. Aerial photograph of the Kusatsu-Shirane volcano taken on 30 October 2021.

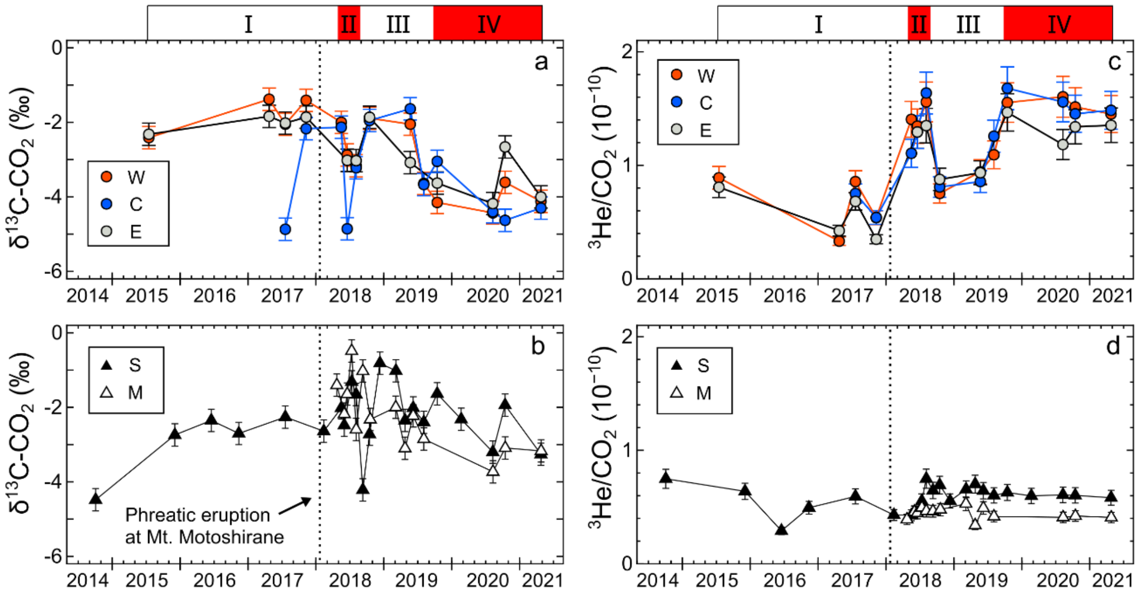

Fig. S2. Temporal variations in $\delta^{13}C\text{-}CO_2$ values and $^3He/CO_2$ ratios of fumarolic gases at the Kusatsu-Shirane volcano. (a) $\delta^{13}C\text{-}CO_2$ values measured at the W, C, and E fumaroles in the Kitagawa geothermal area. (b) $\delta^{13}C\text{-}CO_2$ values measured at the Sesshogawara (S) and Manza (M) fumaroles. (c) $^3He/CO_2$ ratios measured at the W, C, and E fumaroles. (d) $^3He/CO_2$ ratios measured at the S and M fumaroles. The Roman numerals above the panels (a) and (c) are periods defined by the average $(^3He/^4He)_{corr}$ ratios of the three Kitagawa samples (see text for details). Dotted lines indicate 23 January 2018, when a phreatic eruption occurred at Mt. Motoshirane.
